# Hybrid Gene Selection Algorithm for Cancer Classification Using Nuclear Reaction Optimization (NRO)

**DOI:** 10.3390/cimb47090683

**Published:** 2025-08-25

**Authors:** Shahad Alkamli, Hala Alshamlan

**Affiliations:** Department of Information Technology, College of Computer and Information Sciences, King Saud University, P.O. Box 51178, Riyadh 11543, Saudi Arabia; 444203620@student.ksu.edu.sa

**Keywords:** gene selection, microarray data, cancer classification, nuclear reaction optimization, metaheuristic algorithms, feature selection, bioinformatics

## Abstract

Microarray gene expression data are characterized by high dimensionality and small sample sizes, which complicates cancer classification tasks. To address these challenges, this study proposes a hybrid gene selection approach that integrates a filter-based dimensionality reduction method with a metaheuristic optimizer. Specifically, the method applies the F-score statistical filter to rank and reduce gene features, followed by Nuclear Reaction Optimization (NRO) to refine the selection. This combination is referred to as the F-score-based Nuclear Reaction Optimization method or F-NRO. The performance of F-NRO was evaluated on six publicly available microarray cancer datasets (Colon, Leukemia1, Leukemia2, Lung, Lymphoma, and SRBCT) using Support Vector Machines (SVMs) and Leave-One-Out Cross-Validation (LOOCV). Comparative analysis against several existing hybrid gene selection algorithms demonstrates that F-NRO achieves high classification accuracy, including perfect accuracy on five datasets, using compact gene subsets. These results suggest that F-NRO is an effective and interpretable solution for gene selection in cancer classification tasks.

## 1. Introduction

Cancer persists as a significant worldwide health issue, requiring ongoing innovation in diagnostic techniques [1]. The advent of microarray technology has considerably progressed molecular oncology by allowing the concurrent assessment of thousands of gene expression levels, hence facilitating in the identification of biomarkers for diverse cancer types [2]. Despite these advancements, microarray datasets generally exhibit high dimensionality, characterized by a gene count that significantly surpasses the number of patient samples, resulting in issues such as overfitting, computational inefficiency, and diminished interpretability.

Feature (gene) selection has emerged as a prevalent technique to tackle these difficulties. This technique improves classification accuracy, diminishes noise, and keeps the analysis more manageable by identifying a smaller sample of pertinent genes [3]. In this context, the terms “feature” and “gene” are synonymous, as each feature denotes the expression level of a gene. A range of strategies has been investigated for this task, encompassing several metaheuristic optimization techniques, as discussed in our prior work [4].

A recent work presented Nuclear Reaction Optimization (NRO) [5] as an innovative metaheuristic approach for gene selection in microarray-based cancer classification tasks. The study illustrated the independent efficacy of NRO in identifying useful gene subsets without prior dimensionality reduction across many benchmark datasets [6]. This method underscored the inherent capabilities of NRO, although it also raised the question of whether preprocessing techniques may further augment its performance. The findings also underscored the superior performance of Support Vector Machines (SVMs) compared with alternative classifiers like k-Nearest Neighbors (k-NNs), prompting our sole utilization of SVMs in the present study.

Building on these previous discoveries, this research presents a hybrid gene selection framework that utilizes various filter-based dimensionality reduction strategies (Information Gain, F-score, ReliefF, and mRMR) as preprocessing steps before NRO. These statistical filters instantly evaluate the significance of each gene with regard to class labels, enabling us to diminish the dataset’s dimensionality prior to using the optimization technique. This not only optimizes the search space but also improves computational efficiency and model efficacy.

The filtered gene sets are subsequently refined utilizing the NRO algorithm, and classification performance is assessed employing an SVM. This method integrates rapid, interpretable filtering techniques with a resilient metaheuristic optimizer to provide high-accuracy gene selection, enhancing scalability and generalizability.

The subsequent sections of this work are structured as follows: Section 2 outlines the datasets, preprocessing procedures, and the implementation technique. Section 3 discusses experimental findings and comparative assessment. Ultimately, Section 4 concludes with essential discoveries and recommendations for subsequent research.

## 2. Materials and Methods

**Figure 1:**
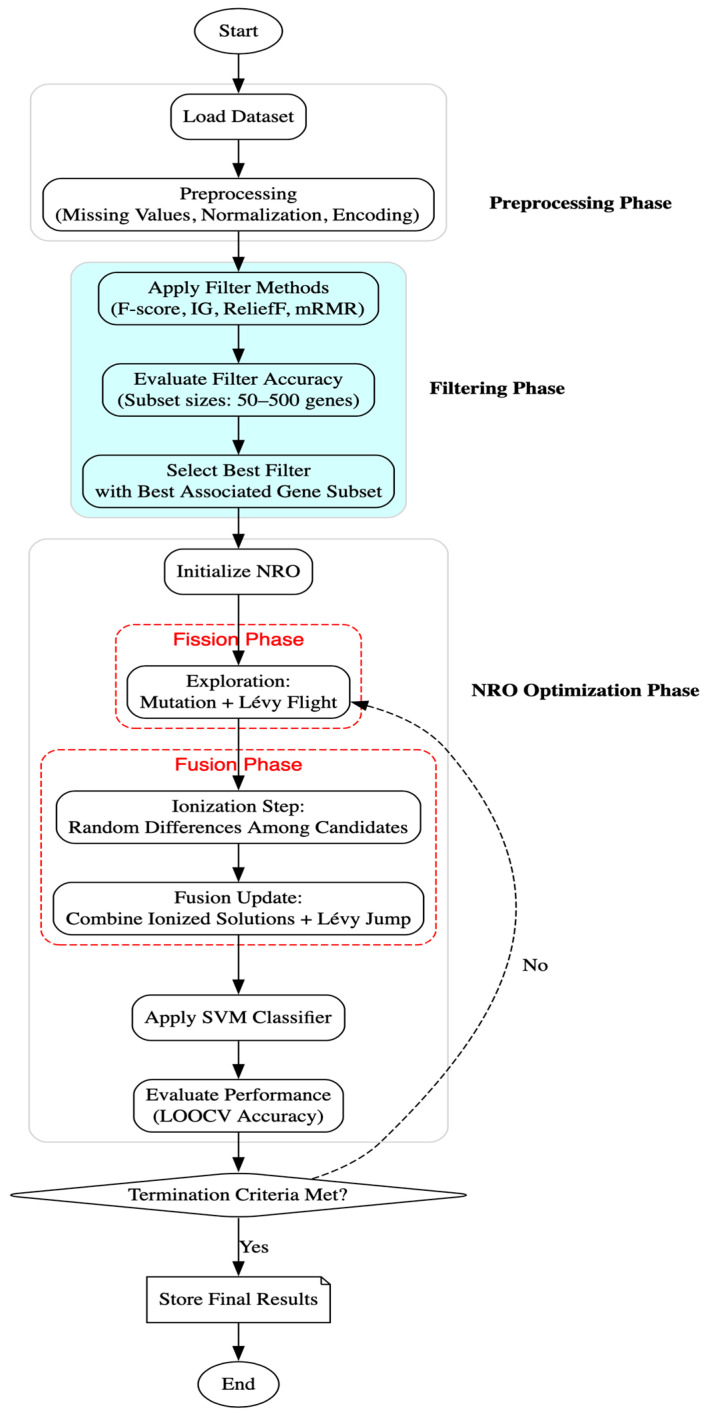
Flowchart of the methodology.

This study explores a hybrid approach that combines filter-based dimensionality reduction with the Nuclear Reaction Optimization (NRO) algorithm for gene selection in cancer classification. The methodology involves dataset preprocessing, application of statistical filters (Information Gain using mutual information and F-score using ANOVA), optimization using NRO, classification via Support Vector Machines (SVMs), and fitness evaluation. The implementation was carried out using Python (version 3.x), employing numpy and pandas for data handling; scipy.io.arff for loading microarray datasets; and scikit-learn for feature filtering (f_classif), scaling, and classification. The NRO algorithm was implemented from scratch, incorporating its mathematical foundations, including nuclear fission and fusion processes, Lévy flight-based step size adjustments, and mutation-based mechanisms for optimizing gene subsets. Figure 1 illustrates the complete workflow, from preprocessing and dimensionality reduction to optimization and performance evaluation.

### 2.1. Dataset and Preprocessing

To evaluate the effectiveness of the proposed hybrid gene selection framework, we utilized six well-known microarray cancer datasets, covering both binary and multiclass classification problems. These datasets are commonly adopted in bioinformatics research to benchmark gene selection and classification techniques. All datasets were obtained from the GEMS system portal (http://www.gems-system.org/, accessed on 15 February 2025). SRBCT [7], Lymphoma [8], and Leukemia2 [9] represent multiclass datasets, while Lung [10], Colon [11], and Leukemia1 [12] are binary-class datasets.

Table 1 provides a summary of each dataset, including the number of classes, samples, and total genes. This selection offers a diverse set of testing conditions, which helps assess the robustness and generalizability of the proposed method.

All datasets were preprocessed to ensure compatibility with machine learning algorithms. Missing values were found only in the Lymphoma dataset, accounting for approximately 4.91% of the total data matrix (12,264 out of 249,612 values). These missing entries were distributed across 2796 of the 4026 genes. Given the relatively low proportion and the wide distribution, the impact on overall data quality was minimal. To preserve data structure without introducing significant bias, missing values were replaced using mean imputation, in which each missing entry was filled with the average expression value of its corresponding gene.

To eliminate differences in scale across gene expression values, Z-score normalization was applied to standardize the data, resulting in a mean of zero and unit variance for each gene. Additionally, class labels in multiclass datasets were numerically encoded using label encoding, ensuring compatibility with the classification model. These preprocessing steps prepared the datasets for subsequent stages of gene selection and classification.

### 2.2. Filter-Based Dimensionality Reduction

To reduce the dimensionality of the gene expression datasets before optimization, we initially applied four widely used statistical filtering techniques: F-score, Information Gain (IG), ReliefF, and minimum Redundancy Maximum Relevance (mRMR). Each filter was independently applied to evaluate and rank genes according to their relevance to the target class labels and to reduce the dataset by retaining only the most relevant genes for further optimization.

#### 2.2.1. Information Gain

Information Gain (IG) is a core concept from information theory, widely used in feature selection tasks to quantify the effectiveness of a feature in classifying data. It measures the expected reduction in entropy (the average amount of information needed to classify a sample) after partitioning the dataset based on that feature [13].

Let D be a dataset containing tuples that belong to m distinct classes $\left\{C_{1},C_{2},\ldots ,C_{m}\right\}$. The entropy of dataset D, denoted as $\mathrm{Info}\left(D\right)$, is defined as:

(1) $$\mathrm{Info}\left(D\right)=-\sum_{i=1}^{m}p_{i}\log_{2}\left(p_{i}\right),$$

where $p_{i}$ is the probability that an arbitrary tuple in D belongs to class $C_{i}$, estimated as $\left|C_{i,D}\right|/\left|D\right|$.

Now, suppose a feature A partitions D into v disjoint subsets $\left\{D_{1},D_{2},\ldots ,D_{v}\right\}$, corresponding to the v distinct values $\left\{a_{1},a_{2},\ldots ,a_{v}\right\}$ observed for feature A. The expected entropy after partitioning by feature A is given by:

(2) $${\mathrm{Info}}_{A}\left(D\right)=\sum_{j=1}^{v}\frac{\left|D_{j}\right|}{\left|D\right|}\cdot \mathrm{Info}\left(D_{j}\right),$$

Here, $\frac{\left|D_{j}\right|}{\left|D\right|}$ represents the weight of the j−th partition and $\mathrm{Info}\left(D_{j}\right)$ is the entropy of that subset.

The Information Gain obtained by using attribute A to partition dataset D is:

(3) $$\mathrm{Gain}\left(A\right)=\mathrm{Info}\left(D\right)-{\mathrm{Info}}_{A}\left(D\right),$$

This metric reflects how effectively feature *A* reduces uncertainty in the classification process. A higher value of Gain (*A*) indicates that the feature is more relevant for distinguishing between classes.

In this study, Information Gain is employed as a filter-based method to rank genes prior to optimization with the NRO algorithm. By selecting the top-ranked genes based on their IG values, we reduce the dimensionality of the feature space, thereby improving both computational efficiency and model performance.

#### 2.2.2. F-Score

F-score is a simple yet effective statistical filter method widely used for feature selection, particularly in binary classification problems. It measures the discriminative power of each feature by comparing the variance between class means to the variance within each class. Features with higher F-scores are considered more relevant, as they provide greater separation between the classes [14].

Let *a* dataset D contain n samples and m features, where each sample belongs to one of two classes: positive (+) and negative (−). For a given feature $x_{i}$, let

1. $\mu_{i}^{+}$ be the mean of $x_{i}$ in the positive class;
2. $\mu_{i}^{-}$ be the mean of $x_{i}$ in the negative class;
3. $\mu_{i}$ be the global mean of $x_{i}$ across all samples;
4. $n^{+}$ and $n^{-}$ be the number of samples in the positive and negative classes, respectively.

The F-score of feature $x_{i}$ is computed as follows:

(4) $$F_{i}=\frac{{\left(\mu_{i}^{+}-\mu_{i}\right)}^{2}+{\left(\mu_{i}^{-}-\mu_{i}\right)}^{2}}{\frac{1}{n^{+}-1}\sum_{k=1}^{n^{+}}{\left(x_{k,i}^{+}-\mu_{i}^{+}\right)}^{2}+\frac{1}{n^{-}-1}\sum_{k=1}^{n^{-}}{\left(x_{k,i}^{-}-\mu_{i}^{-}\right)}^{2}}$$

The numerator represents the between-class variance, quantifying how distinct the class means are relative to the overall mean. The denominator captures the within-class variance, reflecting the dispersion of feature values inside each class.

A larger $F_{i}$ implies that feature $x_{i}$ exhibits stronger discrimination between the two classes, making it more useful for classification. Unlike wrapper-based methods, which involve training a classifier, F-score is computationally efficient and independent of any learning algorithm, making it well suited for high-dimensional datasets such as microarray gene expression data [15].

In this study, F-score is employed as a filtering mechanism to preselect the most informative genes before applying the Nuclear Reaction Optimization (NRO) algorithm. This two-stage approach allows for more focused exploration in the optimization phase and contributes to improved classification performance and reduced computational complexity.

#### 2.2.3. ReliefF

ReliefF is a filter-based feature selection method that estimates the importance of each feature based on how well it distinguishes between instances that are close to each other. Unlike univariate filters such as Information Gain and F-score, ReliefF considers feature dependencies by examining the nearest neighbors of each sample. For each randomly selected instance, ReliefF identifies its nearest neighbor from the same class (nearest hit) and from different classes (nearest miss), updating feature weights based on the observed differences. Features that show large differences between different-class neighbors and small differences between same-class neighbors are assigned higher relevance scores [16].

ReliefF is particularly robust against noise, can handle multiclass problems, and captures local dependencies between features, advantages that make it well suited for high-dimensional data like microarray gene expressions.

In this study, ReliefF is employed to rank genes based on their discriminative ability, and its effectiveness is evaluated comparatively with other filters in the Results Section.

#### 2.2.4. Minimum Redundancy Maximal Relevancy (mRMR)

Minimum Redundancy Maximum Relevance (mRMR) is a filter method that selects features by maximizing their relevance to the class labels while minimizing redundancy among the selected features. Relevance and redundancy are both measured using mutual information [17]. The mRMR criterion is defined as follows:

(5) $$\mathrm{mRMR}=\mathrm{MI}\left(f,c\right)-\frac{1}{\left|S\right|}\sum_{s\in S}\mathrm{MI}\left(f,s\right)$$

where $\mathrm{MI}\left(f,c\right)$ is the mutual information between a feature f and the class c, and $\mathrm{MI}\left(f,s\right)$ is the mutual information between feature f and each already-selected feature s.

In this study, mRMR is used to rank genes based on a balance of relevance and redundancy, with its performance compared with that of other filters in subsequent results.

From each ranked list, the top 500 genes (k = 500) were selected to form reduced versions of the original datasets. The value of 500 was chosen after empirical experimentation with various subset sizes (ranging from 50 to 500 genes), aiming to balance the goals of substantial dimensionality reduction and retention of predictive gene information.

The filtering processes were implemented as follows: F-score and Information Gain were computed using the f_classif and mutual_info_classif functions from the scikit-learn library. ReliefF and mRMR were implemented using the reliefF module from skfeature.function.similarity_based and the MRMR module from skfeature.function.information_theoretical_based, respectively. All implementations were adapted to ensure compatibility and consistency across datasets.

The comparative evaluation of the filtering methods, and the subsequent selection of the best-performing filter for integration with the Nuclear Reaction Optimization (NRO) algorithm are discussed in detail in the Results Section.

### 2.3. Applying the Nuclear Reaction Optimization (NRO) Algorithm

In this study, the NRO algorithm was employed as the search method to identify informative subsets of genes from high-dimensional microarray datasets. NRO algorithm is a physics-inspired metaheuristic designed to address optimization challenges by mimicking the natural processes of nuclear fission and fusion [5]. These occurrences illustrate the processes by which atomic nuclei undergo either fission or fusion to release energy.

Many metaheuristic algorithms use a single type of search move throughout the run, which can make them efficient but also more likely to settle into one part of the search space too early. NRO works differently; by switching between two distinct phases (fission to push solutions far apart and explore, and fusion to combine useful parts and refine) it can search widely without losing the ability to fine-tune. The built-in step size adjustment and occasional long jumps mean it can escape from areas that look promising at first but turn out to be sub-optimal. In gene selection, in which relevant features may be scattered among thousands of irrelevant ones, this flexibility helps NRO locate and keep improving diverse, high-quality gene subsets.

#### 2.3.1. Nuclear Fission Phase

In NRO, the nuclear fission process produces new candidate solutions by partitioning an existing solution into smaller elements. This method allows the algorithm to investigate many areas of the solution space, hence minimizing the likelihood of becoming ensnared in a local optimum. The procedure is regulated by the subsequent equation:

(6) $$X_{i}^{Fi}\left\{\begin{array}{c} \mathrm{Gaussian}\left(X_{best},\sigma_{1}\right)+\;\left({rand}_{n}\cdot X_{best}-P_{ne}^{s}\cdot {Ne}_{i}\right),\;\;\;\;if\;rand\;\leq \;P_{\beta },\\ \mathrm{Gaussian}\left(X_{i},\sigma_{2}\right)+\;\left({rand}_{n}\cdot X_{best}-P_{ne}^{e}\cdot {Ne}_{i}\right),\;\;\;\;\;\;\;\;\;if\;rand\;>\;P_{\beta }, \end{array}\right.$$

Here,

5. $X_{i}^{Fi}:$ the novel solution produced during fission;
6. $X_{best}:$ the optimal solution identified to date;
7. $Gaussian\left(X,\sigma \right):$ a random variable produced around X, with σ determining the dispersion;
8. $\sigma_{1},\sigma_{2}$: parameters regulating the extent of exploration (see Equations (7) and (8) below);
9. ${rand}_{n}:$ a random variable introducing variability in the solution;
10. $P_{ne}^{s},P_{ne}^{e}:$ mutation factors determining the scale of adjustments for subaltern and essential fission products, respectively;
11. ${Ne}_{i}:$ heated neutron, calculated as ${Ne}_{i}=\frac{X_{i}}{X_{j}},$ where $X_{i}\;\mathrm{and}\;X_{j}$ are two random solutions;
12. $P_{\beta }:$ the probability governing whether subaltern or essential fission products are produced.

This method ensures a balanced search by generating some solutions near the best one $X_{best}$ for local refinement, while others are positioned farther away to promote broader exploration.

The fission process’s efficiency is affected by the step size, which regulates the degree of divergence of new solutions from existing ones. The step sizes are calculated with the subsequent formula:

(7) $$\sigma_{1}=\left(\frac{\log\left(g\right)}{g}\right)\cdot \left|X_{i}-X_{\mathrm{best}}\right|,$$

(8) $$\sigma_{2}=\left(\frac{\log\left(g\right)}{g}\right)\cdot \left|X_{r}-X_{\mathrm{best}}\right|.$$

Here,

13. g: current generation number; the term $\frac{\log\left(g\right)}{g}$ guarantees a reduction in step sizes as iterations advance;
14. $\left|X_{i}-X_{\mathrm{best}}\right|:$ the distance between the current solution and the best-known solution;
15. $\left|X_{r}-X_{\mathrm{best}}\right|:$ the distance between a random solution and the best-known solution.

Initially, substantial step sizes facilitate the exploration of the solution space. Subsequently, the step sizes diminish, enabling the algorithm to narrow down on the most effective solutions and achieve an optimal outcome.

Mutation factors are embedded in the fission equation to either intensify or broaden the search, helping prevent premature convergence and promoting efficient optimization. The factors are defined as follows:

(9) $$P_{\mathrm{ne}}^{s}=\mathrm{round}\left(rand+1\right),$$

(10) $$P_{\mathrm{ne}}^{e}=\mathrm{round}\left(rand+2\right),$$

Here,

16. rand: a random number uniformly distributed between 0 and 1;
17. The integer rounding guarantees discrete adjustment levels for the mutation process.

#### 2.3.2. Nuclear Fusion Phase

The nuclear fusion process improves solutions by combining promising candidates and functions through two primary sub-phases: ionization and fusion.

In the ionization sub-phase, solutions are adjusted by utilizing the disparities among randomly selected candidates. This modification is executed utilizing the subsequent formula:

(11) $$X_{i,d}^{Ion}\left\{\begin{array}{c} X_{r1,d}^{Fi}\;+\;rand\;\cdot \;\left(X_{r2,d}^{Fi}-X_{i,d}^{Fi}\right),\;\;if\;rand\;\leq \;0.5,\\ X_{r1,d}^{Fi}\;-\;rand\;\cdot \;\left(X_{r2,d}^{Fi}-X_{i,d}^{Fi}\right)\;\;\;\;if\;rand\;>\;0.5, \end{array}\right.$$

Here,

18. $X_{r1,d}^{Fi},X_{r2,d}^{Fi}$: components of two randomly selected fission solutions;
19. $X_{i,d}^{Fi}:$ current solution;
20. rand: random value for diversity.

If the chosen solutions exhibit excessive similarity, Lévy flight [18] is employed to provide significant changes, facilitating the algorithm’s escape from stagnation and enabling exploration of novel regions within the search space:

(12) $$X_{i,d}^{\mathrm{Ion}}=X_{i,d}^{\mathrm{Fi}}+{\left(\alpha ⊗\mathrm{Levy}\left(\beta \right)\right)}_{d}\cdot \left(X_{i,d}^{\mathrm{Fi}}-X_{\mathrm{best},d}^{\mathrm{Fi}}\right),$$

Here,

21. α: a scaling factor controlling the magnitude of jumps;
22. $\mathrm{Levy}\left(\beta \right):$ heavy-tailed random step size, introducing both small and large adjustments;
23. ⊗: indicates element-wise multiplication;
24. $X_{\mathrm{best},d}^{\mathrm{Fi}}$: best-known solution in the dth dimension.

Levy flight is particularly activated when the difference term $X_{r2,d}^{Fi}\;-\;X_{i,d}^{Fi}$ approaches zero, as this indicates a risk of stagnation in the search process.

During the fusion sub-phase, the algorithm merges the advantages of superior solutions to enhance the search process. The fusion process is denoted by the subsequent equation:

(13) $$X_{i}^{\mathrm{Fu}}=X_{i}^{\mathrm{Ion}}+rand\cdot \left(X_{r1}^{\mathrm{Ion}}-X_{\mathrm{best}}\right)+rand\cdot \left(X_{r2}^{\mathrm{Ion}}-X_{\mathrm{best}}\right),$$

Here,

25. $X_{i}^{\mathrm{Fu}}$: refined solution after fusion;
26. $X_{\mathrm{best}}:$ best-known solution guiding the search;
27. $X_{r1}^{\mathrm{Ion}},X_{r2}^{\mathrm{Ion}}:$ ionized solutions selected for comparison;
28. rand: random value for diversity.

The search process may stagnate; Lévy flight is utilized to facilitate random, long-distance hops, enabling the algorithm to escape local optima and persist in exploring novel areas. This mechanism is articulated by the subsequent equation:

(14) $$X_{i}^{\mathrm{Fu}}=X_{i}^{\mathrm{Ion}}+\alpha ⊗\mathrm{Levy}\left(\beta \right)⊗\left(X_{i}^{\mathrm{Ion}}-X_{\mathrm{best}}^{\mathrm{Ion}}\right),$$

Overall, the NRO algorithm utilizes the dynamic interaction between fission and fusion to effectively traverse the search space. Its physics-inspired processes enable adaptive exploration of varied regions while consistently converging on optimal solutions, making it a resilient technique for complicated optimization challenges.

After establishing the underlying equations and operational principles of NRO, the algorithm was then configured for this study. Each candidate solution was expressed as a binary vector in which a value of 1 denoted selection of the corresponding gene and a value of 0 indicated exclusion. This representation allowed the algorithm to operate directly on discrete feature combinations without requiring additional transformation steps.

The population size was set to 500 after exploratory tests with different values. Smaller populations tended to converge prematurely, resulting in insufficient exploration of the search space. Larger populations increased diversity but also led to significantly longer runtimes, particularly for datasets containing several thousand genes, without a consistent improvement in classification accuracy. The selected size provided a balance between maintaining diversity and achieving computational efficiency.

A maximum of 30 generations was used for each run. Preliminary trials showed that most datasets achieved their best performance before this limit, although some benefited from additional iterations. To reduce unnecessary computation when no further progress was expected, an early-stopping rule was applied: the search terminated if there was no improvement in the best fitness value for five consecutive generations. This criterion was determined empirically from pilot experiments in which prolonged stagnation rarely led to performance gains.

Because NRO is stochastic in nature, results can vary across runs. To ensure robust and reproducible performance estimates, each experiment was repeated 30 times. The mean of these repetitions was reported for all evaluation metrics, thereby reducing the influence of random fluctuations.

Parameters that control mutation, step size, and Lévy-flight behavior were set according to the original NRO formulation [5]. These values have been shown to produce a stable balance between exploration in the early search phases and exploitation during later stages. The adaptive step size mechanism enabled the search to begin with broad exploratory moves and then gradually focus on refining promising solutions. Lévy flights were incorporated occasionally to enable large, abrupt moves that help escape local optima.

The complete implementation procedure of the NRO-based gene selection method, incorporating these parameter choices, is summarized in the Algorithm 1 provided below. This pseudocode outlines the initialization process, the iterative search operations, and the stopping criteria as applied in this study.
**Algorithm 1** Nuclear Reaction Optimization (NRO) Algorithm for Gene Selection**Require:** Dataset *D*, Population size *N* = 500, Max generations *T* = 30, Early stopping patience *P* = 5 **Ensure:** Optimized subset of gene
▷ **Preprocessing Phase**
1:  Handle missing values (mean imputation) 2:  Normalize features using Z-score 3:  Encode categorical labels▷ **Filter Evaluation Phase**4:  Apply filter methods: F-score, Information Gain, ReliefF, and mRMR 5:  **for** each filter method **do**
6:     **for** each subset size *s* ∈ {50, 100, …, 500} **do** 7:        Select top *s* genes using the filter 8:        Evaluate subset using SVM classifier with LOOCV 9:    **end for** 10:  **end for** 11:  Select best filter method with its best-performing gene subset▷ **NRO Optimization Phase**12:  **for** each subset size *k* ∈ {2, …, 25} **do** 13:      Initialize population of binary vectors of size *k* over selected genes 14:      Set bounds [0, 1], initialize global best solution $X_{\mathrm{best}}$ 15:      Compute initial fitness using SVM + LOOCV 16:      Initialize no improve ← 0▷ Fission Phase: Exploration via perturbation17:      **for** *g* = 1 to *T* **do** 18:        **for** each solution $x_{i}$ **do** 19:           Generate new solutions as per Equation (6) 20:           Adjust step sizes using Equations (7) and (8) 21:           Apply mutation using Equations (9) and (10) 22:        **end for**▷ Fusion Phase: Exploitation with embedded crossover23:        **for** each solution $x_{i}$ **do** 24:           Adjust using ionization (Equation (11)) 25:           **if** solutions are similar **then** 26:             Apply Lévy flight adjustment (Equation (12)) 27:           **end if** 28:           Fuse solutions (Equation (13)) 29:           **if** solutions are still similar **then** 30:             Apply Lévy flight adjustment (Equation (14)) 31:           **end if** 32:        **end for**▷ **Fitness Evaluation and Best Solution Update**33:        **for** each solution $x_{i}$ **do** 34:           Compute LOOCV classification accuracy 35:           **if** $Fitness(x_{i})$ > $Fitness(X_{\mathrm{best}})$ **then** 36:             Update $X_{\mathrm{best}}$ ← $x_{i}$, reset *no_improve* ← 0 37:           **else** 38:             Increment *no_improve* ← *no_improve* + 1 39:           **end if** 40:        **end for** 41:        **if** *no_improve* ≥ P **then** 42:           **break**
43:        **end if** 44:    **end for** 45:  **end for**▷ **Final Output**46:   **return** best gene subset $X_{\mathrm{best}}$


### 2.4. Classification and Fitness Evaluation

The Support Vector Machines (SVMs) were used as the classification model to assess the quality of the gene subsets selected by the optimization algorithm. Based on findings from our previous study and supported by the extensive literature on bioinformatics, SVMs have demonstrated superior performance in high-dimensional gene expression data. Their ability to construct optimal hyperplanes in sparse feature spaces makes them particularly well suited for microarray-based cancer classification tasks.

In this study, SVMs were configured with a linear kernel and a regularization parameter C = 1, providing an effective balance between margin maximization and classification sensitivity. This configuration was applied consistently across all datasets to ensure uniformity in model evaluation.

Fitness was assessed based on classification performance, measured through Leave-One-Out Cross-Validation (LOOCV). LOOCV is a robust evaluation method well suited for datasets with small sample sizes. It operates by using each individual sample once as a test case while the remaining samples form the training set, resulting in a thorough and unbiased estimate of predictive accuracy.

As part of the evaluation process, both 5-fold and 10-fold cross-validation methods were applied to all datasets. These approaches consistently resulted in classification accuracies that were 1–2% lower than those achieved with LOOCV. Although LOOCV required more computational time, often between two and ten times longer depending on the dataset, it provided more reliable performance. Given the small sample sizes, where each instance plays a significant role in learning, LOOCV was selected to reduce bias and improve model stability. Its use is also supported by previous studies in bioinformatics that show that LOOCV enhances generalization and reduces the risk of overfitting in high-dimensional gene expression analysis [19,20,21].

## 3. Results and Discussion

### 3.1. Dimensionality Reduction

**Figure 2:**
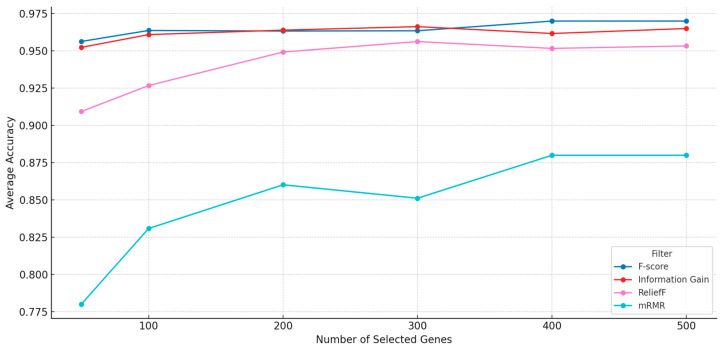
Filters’ average accuracy vs. number of filtered genes.

In the preprocessing phase, four filter methods—F-score, Information Gain (IG), ReliefF, and mRMR—were independently applied to rank genes based on their relevance to the target classes. The performance of each filter was initially assessed across multiple subset sizes (50, 100, 200, 300, 400, and 500 genes) using classification accuracy as the evaluation metric. As shown in Figure 2, F-score achieved the highest classification accuracy across most subset sizes, closely followed by Information Gain. ReliefF and mRMR generally produced lower classification results. To further validate these observations, the gene subsets generated by each filter were also optimized using the NRO algorithm and evaluated for final classification performance. The results confirmed that IG-, ReliefF-, and mRMR-based preprocessing led to lower accuracies after optimization than that obtained using F-score. Based on this evaluation, only F-score was selected for final integration with the NRO algorithm, while the other three were excluded.

### 3.2. F-Score-Based Nuclear Reaction Optimization (F-NRO) Algorithm

This section reports the performance of the hybrid algorithm combining the F-score filter with Nuclear Reaction Optimization (F-NRO) across the six microarray cancer datasets. The evaluation emphasizes best, average, and worst accuracies, supported by precision, recall, F1-score, and confidence intervals, to assess both predictive performance and stability. In the experiments we evaluated every subset size from 2 to 25 genes, and to keep the discussion focused, we tabulated only those subset sizes that changed the predictive metrics, and we stopped reporting once the model reached perfect accuracy for the dataset in question.

As shown in Table 2, in the Colon dataset, classification accuracy rose steadily from ≈92% with two genes to ≈98% once 22 genes were retained. The broad confidence interval at two genes ([88.4%, 89.6%]) narrowed as additional markers were added, signaling reduced variance across the 30 repeated runs. Precision, recall, and the F1-score progressed in near lockstep, which confirms that the added genes improved true-positive recognition without inflating false positives. The largest single gain occurred between 5 and 9 genes, after which returns diminished, suggesting that the 22-gene “sweet spot” is driven more by stabilizing borderline samples than by uncovering a wholly new discriminative signal.

As shown in Table 3, in the Leukemia1 dataset, the model already exceeded 98% accuracy with just two genes and reached 100% once a third gene was introduced; adding a fourth gene did not alter any metric. The vanishingly small 95% confidence interval at three genes (98.7–99.2%) shows the solution is both compact and statistically robust. In practice, a three-gene panel would therefore suffice for clinical discrimination in this binary setting, limiting assay costs while preserving certainty.

According to Table 4, in the Leukemia2 dataset, the trajectory is more gradual: accuracy climbs from 95.8% at two genes to 98.6% at four and five genes and then attains 100% at seven genes. Each additional marker tightens the confidence bounds, indicating that the optimization phase is resolving class overlap rather than merely memorizing samples. The plateau at seven genes validates the early-stopping rule; beyond that point, no statistical improvement would be expected.

From Table 5, it is evident that the Lung data are highly separable: perfect accuracy, precision, and recall were obtained with only a two-gene subset, and the metrics remained invariant when a third gene was added. Because the 95% confidence interval collapses to a point mass, any further enlargement of the gene panel would add complexity without diagnostic benefit.

As presented in Table 6, in the Lymphoma dataset, the hybrid F-NRO pipeline achieved 100% accuracy with two genes and maintained it as a third gene was included. The near-perfect but non-zero confidence range at two genes slightly narrows at three, hinting that the extra marker offers marginal insurance against out-of-sample variability, though at the cost of a marginally longer panel.

As shown in Table 7, because SRBCT comprises four tumor subtypes, the model needed a broader feature set: accuracy improved from 86.8% (two genes) through 93.0% (three) and 96.4% (four) until it reached 100% with a seven-gene subset. Precision and recall followed the same curve, confirming balanced performance across classes. The monotonic rise and eventual plateau reflect the optimization algorithm’s capacity to add only those genes that resolve remaining misclassifications, after which further additions become redundant.

### 3.3. Comparative Analysis

Table 8 contrasts the proposed F-NRO algorithm with seven published hybrid filter–metaheuristic gene-selection algorithms: F-FSAPV, F-FF, Relief-MBO, mRMR-ABC, mRMR-PSO, mRMR-GA, and Co-ABC. Classification accuracy is used as the common metric, and the number of genes retained by each algorithm is given in parentheses. The comparison highlights how well each approach balances predictive performance with subset compactness across six publicly available microarray cancer datasets.

F-NRO delivers the most complete and accurate coverage of all six benchmarks: it attains perfect 100% accuracy on five datasets (Leukemia 1, Leukemia 2, Lung, Lymphoma, SRBCT) and posts the highest reported score on Colon (98.39%), whereas every competing hybrid either leaves gaps in its evaluation suite or falls short on at least one dataset. Co-ABC equals F-NRO’s five perfect results but trails on Colon (96.77%), while mRMR-ABC matches the five perfect scores yet never surpasses F-NRO and relies on much larger subsets (up to 20 genes). Relief-MBO is exceptionally parsimonious and nearly ties F-NRO on Colon (98.20% with just three genes) but is unreported for Lung and Leukemia 2. F-FSAPV and F-FF each achieve several perfect accuracies but omit multiple datasets and are lower on Colon; mRMR-PSO and mRMR-GA cover even fewer tasks and yield their best results with double-digit gene counts.

Taken together, the comparison underscores F-NRO’s position as the most consistently accurate of the evaluated filter-based hybrids, yet it also pinpoints clear avenues for refinement. The method still falls just short of the 100 percent ceiling on the Colon dataset, indicating that an additional exploitation mechanism is needed to push accuracy to perfection there. At the same time, although F-NRO attains flawless predictions on Leukemia 2 and SRBCT, it achieves this with seven-gene panels; a more aggressive gene-pruning step could trim those subsets while preserving the perfect scores. Addressing these two points would complete the performance profile: perfect accuracy across all six benchmarks and minimal gene signatures across the board.

## 4. Conclusions

This study introduced a hybrid gene selection framework that combines the F-score filtering method with Nuclear Reaction Optimization (NRO), referred to as F-score-based Nuclear Reaction Optimization (F-NRO), for effective classification of cancer using microarray gene expression data. The proposed method demonstrated high accuracy across six benchmark datasets, achieving perfect classification performance in five cases while maintaining concise gene subsets. Compared with existing hybrid approaches, F-NRO showed a strong balance between predictive power and subset compactness. From a biological perspective, the ability to obtain high accuracy with small gene sets suggests that the selected features capture key discriminative signals that may correspond to meaningful biomarkers. The stability of results across diverse datasets indicates that F-NRO is robust to differences in microarray platforms and cancer types, a property that could be valuable for developing broadly applicable diagnostic models. Statistically, achieving this level of performance with reduced dimensionality highlights the method’s capacity to mitigate overfitting while retaining generalizability.

Future work will focus on further reducing the number of selected genes for datasets such as Colon, SRBCT, and Leukemia2, for which slightly larger subsets were needed. Potential directions include hybridizing NRO with other metaheuristic algorithms to enhance exploration and convergence or embedding additional mechanisms into the NRO fusion phase, since fusion is responsible for refining and combining promising solutions. The fission phase already provides strong exploration by diversifying the search space, whereas enhancing fusion primarily strengthens exploitation while preserving this exploratory capability, as we explored in our subsequent work [31]. Additionally, biological validation of the selected genes could help confirm their relevance and support clinical translation.

## Figures and Tables

**Table 1:** Statistics of microarray cancer datasets.

Microarray Dataset	Classes	Samples	Total Genes
Colon [11]	2	62	2000
Leukemia1 [12]	2	72	7129
Leukemia2 [9]	3	72	7129
Lung [10]	2	96	7129
Lymphoma [8]	3	62	4026
SRBCT [7]	4	83	2308

**Table 2:** The performance of the proposed F-NRO algorithm for the Colon dataset.

Dataset	Total Genes	Filtered Genes	Selected Genes	Accuracy			Precision	Recall	F1-Score	CI (95%)
				Best	Average	Worst				
Colon	2000	500	2	91.94%	88.98%	83.87%	92.58%	91.94%	92.04%	[88.38%, 89.57%]
			3	91.94%	90.59%	87.10%	92.83%	91.94%	91.97%	[90.20%, 90.98%]
			4	93.55%	91.51%	87.10%	93.62%	93.55%	93.55%	[91.12%, 91.89%]
			5	95.16%	92.53%	87.10%	95.26%	95.16%	95.18%	[92.04%, 93.01%]
			9	96.77%	94.35%	90.32%	97.04%	96.77%	96.80%	[93.94%, 94.77%]
			22	98.39%	94.84%	91.94%	98.43%	98.39%	98.38%	[94.38%, 95.30%]

**Table 3:** The performance of the proposed F-NRO algorithm for the Leukemia1 dataset.

Dataset	Total Genes	Filtered Genes	Selected Genes	Accuracy			Precision	Recall	F1-Score	CI (95%)
				Best	Average	Worst				
Leukemia1	7129	500	2	98.61%	98.29%	94.44%	98.64%	98.61%	98.60%	[98.06%, 98.51%]
			3	100%	98.94%	95.83%	100%	100%	100%	[98.71%, 99.16%]
			4	100%	99.40%	95.83%	100%	100%	100%	[99.14%, 99.66%]

**Table 4:** The performance of the proposed F-NRO algorithm for the Leukemia2 dataset.

Dataset	Total Genes	Filtered Genes	Selected Genes	Accuracy			Precision	Recall	F1-Score	CI (95%)
				Best	Average	Worst				
Leukemia2	7129	500	2	95.83%	92.36%	84.72%	95.93%	95.83%	95.84%	[91.89%, 92.83%]
			3	95.83%	94.49%	88.89%	96.28%	95.83%	95.93%	[94.09%, 94.89%]
			4	98.61%	96.06%	91.67%	98.75%	98.61%	98.64%	[95.59%, 96.54%]
			5	98.61%	96.90%	91.67%	98.75%	98.61%	98.64%	[96.52%, 97.28%]
			6	98.61%	97.45%	93.06%	98.75%	98.61%	98.64%	[97.18%, 97.73%]
			7	100%	98.19%	93.06%	100%	100%	100%	[97.86%, 98.53%]

**Table 5:** The performance of the proposed F-NRO algorithm for the Lung dataset.

Dataset	Total Genes	Filtered Genes	Selected Genes	Accuracy			Precision	Recall	F1-Score	CI (95%)
				Best	Average	Worst				
Lung	7129	500	2	100%	100%	98.96%	100%	100%	100%	100% (constant)
			3	100%	100%	100%	100%	100%	100%	100% (constant)

**Table 6:** The performance of the proposed F-NRO algorithm for the Lymphoma dataset.

Dataset	Total Genes	Filtered Genes	Selected Genes	Accuracy			Precision	Recall	F1-Score	CI (95%)
				Best	Average	Worst				
Lymphoma	4026	500	2	100%	98.94%	95.45%	100%	100%	100%	[98.68%, 99.20%]
			3	100%	99.95%	96.97%	100%	100%	100%	[99.85%, 100.05%]

**Table 7:** The performance of the proposed F-NRO algorithm for the SRBCT dataset.

Dataset	Total Genes	Filtered Genes	Selected Genes	Accuracy			Precision	Recall	F1-Score	CI (95%)
				Best	Average	Worst				
SRBCT	2308	500	2	86.75%	81.29%	73.49%	87.42%	86.75%	86.84%	[80.66%, 81.91%]
			3	93.98%	90.00%	80.72%	94.21%	93.98%	93.99%	[89.37%, 90.63%]
			4	96.39%	93.21%	83.13%	96.77%	96.39%	96.39%	[92.64%, 93.79%]
			5	97.59%	94.98%	87.95%	97.77%	97.59%	97.61%	[94.39%, 95.57%]
			6	98.80%	95.86%	89.16%	98.90%	98.80%	98.81%	[95.40%, 96.33%]
			7	100%	96.71%	90.36%	100%	100%	100%	[96.18%, 97.23%]

**Table 8:** Classification accuracy and gene subset sizes across hybrid gene selection methods.

Algorithm	Colon	Leukemia1	Leukemia2	Lung	Lymphoma	SRBCT
F-NRO	98.39% (22)	100% (3)	100% (7)	100% (2)	100% (2)	100% (7)
F-FSAPV [22]	96.9% (7)	100% (3)	-	-	-	100% (5)
F-FF [23]	94.3% (15)	100% (5)	97.8% (10)	100% (2)	-	100% (8)
Relief-MBO [24]	98.2% (3)	99.45% (5)	-	-	99.64% (3)	99.87% (6)
mRMR-ABC [25]	96.77% (15)	100% (14)	100% (20)	100% (8)	100% (5)	100% (20)
mRMR-PSO [26]	90.32% (10)	100% (18)	-	-	-	-
mRMR-GA [27]	-	-	-	100% (15)	95% (5)	-
Co-ABC [28]	96.77% (9)	100% (3)	100% (6)	100% (2)	100% (2)	100% (4)
HHO-GRASP [29]	93.88% (7)	-	-	-	-	-
PCC-GA [30]	91.94% (29)	-	100% (35)	97.54% (42)	100% (39)	100% (20)

## Data Availability

The data presented in this study are available on request from the corresponding author.
